# Time-Varying Respiratory System Elastance: A Physiological Model for Patients Who Are Spontaneously Breathing

**DOI:** 10.1371/journal.pone.0114847

**Published:** 2015-01-22

**Authors:** Yeong Shiong Chiew, Christopher Pretty, Paul D. Docherty, Bernard Lambermont, Geoffrey M. Shaw, Thomas Desaive, J. Geoffrey Chase

**Affiliations:** 1 Department of Mechanical Engineering, University of Canterbury, Christchurch, New Zealand; 2 Department of Intensive Care, Christchurch Hospital, Christchurch, New Zealand; 3 GIGA Cardiovascular Science, University of Liege, Liege, Belgium; Technion—Israel Institute of Technology, ISRAEL

## Abstract

**Background:**

Respiratory mechanics models can aid in optimising patient-specific mechanical ventilation (MV), but the applications are limited to fully sedated MV patients who have little or no spontaneously breathing efforts. This research presents a time-varying elastance (*E_drs_*) model that can be used in spontaneously breathing patients to determine their respiratory mechanics.

**Methods:**

A time-varying respiratory elastance model is developed with a negative elastic component (*E_demand_*), to describe the driving pressure generated during a patient initiated breathing cycle. Data from 22 patients who are partially mechanically ventilated using Pressure Support (PS) and Neurally Adjusted Ventilatory Assist (NAVA) are used to investigate the physiology relevance of the time-varying elastance model and its clinical potential. *E_drs_* of every breathing cycle for each patient at different ventilation modes are presented for comparison.

**Results:**

At the start of every breathing cycle initiated by patient, *E_drs_* is < 0. This negativity is attributed from the *E_demand_* due to a positive lung volume intake at through negative pressure in the lung compartment. The mapping of *E_drs_* trajectories was able to give unique information to patients’ breathing variability under different ventilation modes. The area under the curve of *E_drs_* (*AUCE_drs_*) for most patients is > 25 *cmH_2_Os/l* and thus can be used as an acute respiratory distress syndrome (ARDS) severity indicator.

**Conclusion:**

The *E_drs_* model captures unique dynamic respiratory mechanics for spontaneously breathing patients with respiratory failure. The model is fully general and is applicable to both fully controlled and partially assisted MV modes.

## Introduction

Estimation of patient-specific respiratory mechanics has shown promising results in optimising mechanical ventilation (MV) on a patient-specific basis [1, 2]. However, for spontaneously breathing (SB) patients, additional equipment or invasive clinical manoeuvres are required to determine the patient’s true respiratory mechanics [3, 4]. In particular, the patient’s own breathing effort obscures model-based observation of the mechanics of the sedated, passive lung [5, 6]. Thus, estimating respiratory mechanics to guide MV is currently limited to patients who are fully sedated, and is often less reliable when the patient is semi-conscious, awake and breathing spontaneously [2, 7, 8]. This issue significantly limits the use of model-based methods, as more patients are ventilated with partial ventilation or SB MV modes [9–15].

The respiratory system can be modelled differently, ranging from a simple single compartment lung model to a complex airway branching model [1, 16–19]. Complex models can describe the respiratory system in more detail compared to simpler models. However, these complex models often requires additional clinical protocols and/or invasive measurement and they are less clinically feasible. Docherty et al. [20] noted that an increase of model parameterisation also increases parameter trade-off, and potentially limits the parameter estimation accuracy and predictive capability of the model. Thus, it is important to have a simple model that can capture the fundamental respiratory mechanics of spontaneous breathing patients without added clinical burdens.

Currently, estimation of bedside respiratory mechanics of SB patients remains using a simpler modelling approach [4, 8] and oesophageal pressure measurements are used to eliminate the impact of the patient’s own inspiratory effort on the estimated respiratory mechanics [3, 4, 8] to titrate therapy. However, the application of oesophageal pressure to fully represent patient-specific pleural pressure changes, and spontaneous breathing effort remains widely debated [3, 21]. Thus, there is a need of a method and measurable to describe these pressure changes in the pleural space while maintaining the physiological relevance of the respiratory model.

In this research, a time-varying respiratory elastance model for SB patients is presented. More specifically, a conventional single compartment lung model [1] is extended to capture and provide more in-depth and specific understanding of lung physiology and mechanics for SB patients. The model has been used in several studies and captured known effects such as falling elastance when alveoli opening pressures are exceeded, changes in elastance after a recruitment manoeuvre, and changes in elastance with PEEP [22, 23]. In particular, a physiological construct, ‘negative elastance’ is introduced to capture patients-specific breathing effort. Such a capability, without the requirement of additional invasive measurements would improve and dramatically extend the application of respiratory mechanics to titrate MV care to all respiratory patients.

## Methodology

### Spontaneously Breathing Respiratory Model

The time-varying elastance is derived from the conventional single compartment model. The single compartment model equation describing patient-specific respiratory mechanics during controlled positive pressure ventilation [2] without the influence of offset pressure is defined:

$$P_{aw}\left(t\right)=P_{resistance}\left(t\right)+P_{elastance}\left(t\right)$$ (1)

$$P_{aw}\left(t\right)=R_{rs}\times Q\left(t\right)+E_{lung}\times V\left(t\right)$$ (2)

Where, *P_aw_* is the airway pressure, *P_resistance_* is the pressure drop due to airway resistance, *P_elastance_* is the pressure contributed to the elastic component of the lung, *t* is the time, *R_rs_* is the conducting airway resistance, *Q* is the flow and *E_lung_* is the lung elastance and *V(t)* is the air volume entering the lung (Tidal volume). However, this model only yields reasonable parameter estimates for patients who are fully sedated under controlled ventilation modes [2].

During partially assisted ventilation, when patients are actively participating in breathing, there is an additional pressure component in the right hand side of Equation (1) to account for the pressure changes in the pleural space. This pressure component is known as pleural pressure (*P_pl_*) or driving pressure (*P_drive_*) as shown in Equation (3).

$$P_{aw}\left(t\right)-P_{pl}\left(t\right)=P_{resistance}\left(t\right)+P_{elastance}\left(t\right)$$ (3)

However, *P_pl_* can only be estimated with the use of oesophageal pressure with relatively low reliability [3, 21]. In the time-varying elastance model, the *P_pl_* is divided into two pressure components: 1) constant chest wall pressure (*P_chest_*) and 2) a variable demand pressure (*P_demand_*) as shown in Equation (4).

$$P_{pl}\left(t\right)=P_{chest}\left(t\right)+P_{demand}\left(t\right)$$ (4)

*P_chest_* is a patient-specific constant pressure in the chestwall that is dependent of the patient weight and position. *P_demand_* is the variable pressure changes dependant on patient inspiratory effort or amount of intercostal muscles and diaphragm movement. A higher *P_demand_* will thus indicate higher patient inspiratory effort, or vice-versa. Substituting Equation (3) into (2) will thus give:

$$P_{aw}\left(t\right)=P_{resistance}\left(t\right)+P_{elastance}\left(t\right)+P_{chest}\left(t\right)+P_{demand}\left(t\right)$$ (5)

Noted that both *P_chest_* and *P_demand_* are attributed on the air volume entering to the lung. Thus, both these pressure components can be represented by an elastic property and air volume. Using this assumption, Equation (5) can be modified into:

$$P_{aw}\left(t\right)=R_{rs}\times Q\left(t\right)+E_{lung}\times V\left(t\right)+E_{chest}\times V\left(t\right)+E_{demand}\times V\left(t\right)$$ (6)

$$P_{aw}\left(t\right)=R_{rs}\times Q\left(t\right)+\left(E_{lung}+E_{chest}+E_{demand}\right)\times V\left(t\right)$$ (7)

To maintain structural identifiably of Equation (7) while maintaining its physiological relevance, *E_lung_*, *E_chest_* and *E_demand_* are lumped into one time-varying respiratory elastance, *E_drs_(t)*.

$$E_{drs}\left(t\right)=E_{chest}\left(t\right)+E_{demand}\left(t\right)+E_{lung}\left(t\right)$$ (8)

$$P_{aw}\left(t\right)=R_{rs}\times Q\left(t\right)+E_{drs}\left(t\right)\times V\left(t\right)$$ (9)

Where:

- *E_lung_*, lung elastance—A time-varying measure of the elastic properties of the lung or the collection of alveoli. *E_lung_* decreases if overall alveoli recruitment outweighs the pressure build-up. *E_lung_* will increase if the overall alveoli are stretched with lesser or no further recruitment [22, 23]. Thus, *E_lung_* is the representation of true mechanics that captures the patient-specific response to MV in each breathing cycle and thus provides an indication of the patient disease state.
- *E_chest_*, chest elastance—The elastic properties of the chest wall, including the rib cage, and the intercostal muscles. This elastance subcomponent can be assumed not to vary with disease-state and is thus a patient-specific constant [24].
- *E_demand_*, demand elastance—Represents the patient-specific inspiratory demand, which varies depending on patient-specific and breath-specific effort. The value of *E_demand_* can be negative (*E_demand_* < 0), as it represents the reduced apparent elastance due to the patient’s inspiratory effort creating a pressure reduction in the pleural space to allow negative pressure ventilation. The negative *E_demand_* proposed in this study is a construct, to capture this negative pressure changes that contribute the increasing lung volume.

**Fig 1:**
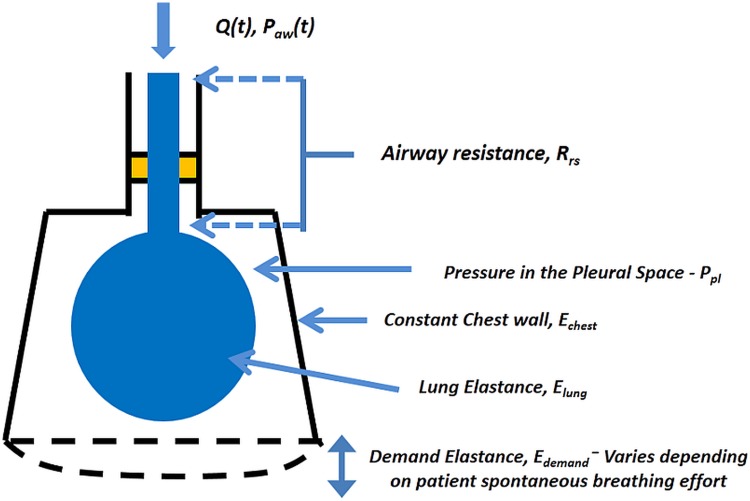
The measured airway pressure consists of 4 pressure components: 1) Pressure drop due to airway resistance (*P_rs_*); 2) pressure in the lung compartments (*Plung(t)* = *Elung(t) ×V(t)*); 3) A constant chest wall pressure, *P_chest_* = *E_chest_(t)×V(t)*; and 4) Demand pressure, *P_demand_* = *E_demand_(t)×V(t)*.

A schematic representation of this model is shown in Fig. 1.

*E_lung_* and *E_chest_* describe the elastance of the patient’s lungs and chest cavity. These values are always positive. However, *E_demand_* represents the change in elastance due to patient-specific breathing effort and is thus negative. In particular, when trying to breathe, the human diaphragm contracts and intercostal muscles move the rib cage upwards and increases the volume of the chest compartment. This increase, creates a negative pressure gradient that draws air into the lungs. During inspiration, *Q* is positive, with increasing *V*. Thus, from Equation (3), the negative pressure from pleural space will result in ‘negative’ values for *E_demand_* (*E_demand_* < 0). As patient demand aids the breathing effort, the effective overall pressure, as seen at the airway, is therefore reduced.

In any given breathing cycle, the time-varying *E_drs_* of Equation (9) captures all three elastance components together. It is important to note that *E_drs_* is a combine effective elastance. It is assessed as the change in pressure for a given tidal volume of flow. Thus, lower effective elastance implies less risk of lung damage [22, 25].

### Data Analysis

To investigate the concept of time-varying *E_drs_*, retrospective data from 22 partially ventilated patients who were ventilated using pressure support (PS) mode and neurally adjusted ventilator assist (NAVA) [26] were studied. In each mode, the airway pressure (*P_aw_*), flow (*Q*) and the electrical diaphragmatic signal (*Eadi*) were recorded. After written informed consent was obtained, the patient was first ventilated using PS for 20 minutes before switching to NAVA for another 20 minutes. The NAVA gain was set to give the same level of pressure support as in the PS mode. The detailed clinical protocol and data acquisition procedure can be found elsewhere [26, 27]. The study protocol and consent procedure was approved by the Ethics committee of the participating hospitals (University Hospital of Geneva (Switzerland) and Cliniques Universitaires St-Luc (Brussels, Belgium)).

In this study, the airway resistance (*R_rs_*) is set as a constant (5 *cmH_2_Os/l*) based on a realistic physiological range [22]. Using this assumption, the *E_drs_* can be modelled as shown in Equation (10) [23]. As a combine effect of all 3 elastance components, any variations of *E_drs_* trajectory can be attributed to changes in *E_lung_*, *E_chest_* and *E_demand_*, while the assumed constant airway resistance allows direct comparison between different ventilation modes for one patient.

$$E_{drs}\left(t\right)=\left(P_{aw}\left(t\right)-R_{rs}\times Q\left(t\right)\right)/V\left(t\right)$$ (10)

**Mapping *E_drs_* Trajectories**. During partial ventilation, the *E_drs_* trajectory during a breath depends on patient inspiratory demand. In addition, the inspiratory time for every breathing cycle is different, and demand is patient-specific and breath-specific. To allow equal visualisation for all *E_drs_* trajectories, the inspiratory time (*Ti*) is normalised and can be interpreted as 0~100% of the inspiratory part of the specific breathing cycle.

Arranging each breathing cycle’s *E_drs_* trajectory, such that it is bounded by the *E_drs_* of the preceding breath and the subsequent breath, leads to a three-dimensional, time-varying, breath-specific *E_drs_* surface (*E_drs_* mapping). This surface allows the effect of changes in ventilator settings on *E_drs_* to be visualised directly. It also clearly shows the breath-to-breath variability together with the effective elastance for each patient and MV mode, allowing them to be quantified [23].

**Assessing *E_drs_* Trajectories and *E_drs_* Area Under the Curve (*AUCE_drs_*)**. For each MV mode, the 5^th^, 25^th^, 50^th^, 75^th^ and 95^th^ percentile of all *E_drs_* trajectories for each patient are presented along with the corresponding airway pressure, lung volume and *Eadi*. For every *E_drs_* trend (the 5^th^, 25^th^, 50^th^, 75^th^ and 95^th^ percentile), the area under curve is calculated (*AUCE_drs_*).

When *E_drs_* < 0, there is effectively ‘no harm’ done to the patient, because any pressure or flow applied is due to the patient’s initial state or demand. However, when a smaller negative *E_drs_* is observed, it indicates that either weak demand or inability of the patient to create significant negative pressure. These cases are of clinical concern, so a less negative *E_drs_* would merit clinical investigation and intervention.

The *E_drs_* between NAVA and PS are compared per-patient, so the patient is his/ her own control. A Kolmogorov-Smirnov test and a signed Wilcoxon ranksum test are used for significance testing. A value of p < 0.05 indicates the NAVA median *E_drs_* is significantly different than that of the PS. The *AUCE_drs_* [22], defined as the area under the curve of *E_drs_* values between 0.3–1.0 seconds of normalised inspiration is also calculated and compared between modes.

## Results

**Fig 2:**
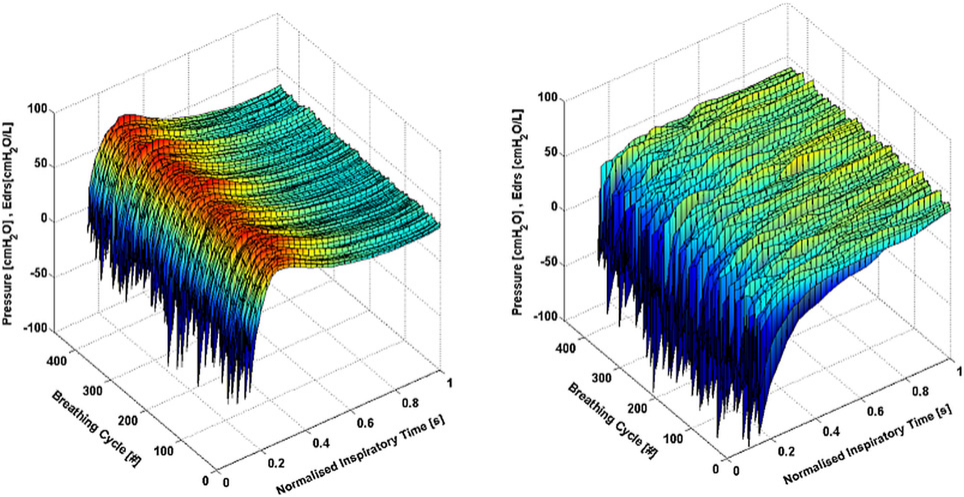
Mapping of *E_drs_* trajectory for Patient 9 during PS (Left) and NAVA (Right). The magnitude of *E_drs_* scale is set identical for direct comparison.

**Fig 3:**
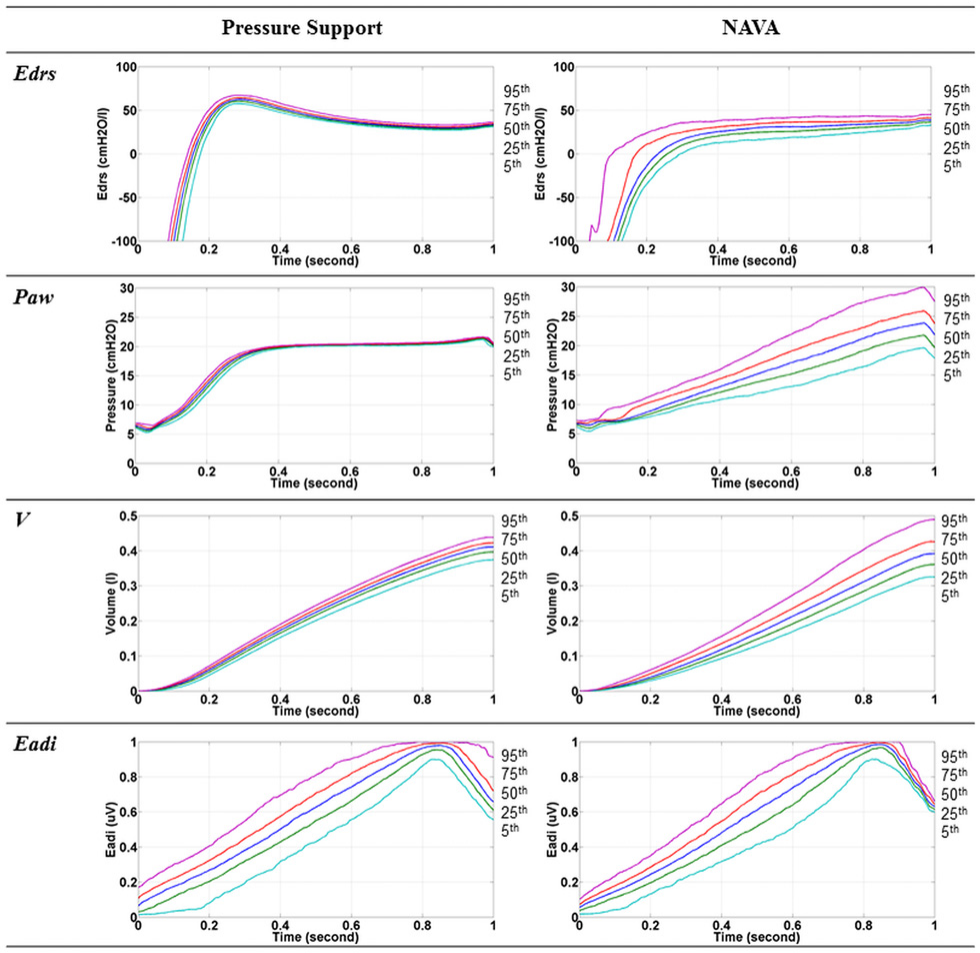
Time-varying *E_drs_*, pressure, volume and electrical diaphragm activity (*Eadi)* curves for Patient 9 during PS (left) and NAVA (Right). The lines indicate the 5^th^ (Light blue), 25^th^ (Green) 50^th^ (Blue), 75^th^ (Red) and 95^th^ (Pink) percentile of all breathing cycles. The sequence where 5^th^, 25^th^, 50^th^, 75^th^ and 95^th^ percentile line occurs is labelled at the side of each figure.

Fig. 2 shows the *E_drs_* trajectories mapping for every breathing cycle (Patient 9). For the same patient, the 5^th^, 25^th^, 75^th^ and 95^th^ percentile of *E_drs_* trajectory, *P_aw_*, *V*, and *Eadi* curves during PS and NAVA is shown in Fig. 3. It should be noted in Fig. 3 that the range of *Eadi* is the same for PS and NAVA, but NAVA has lower and more variable *E_drs_* and more variable *V* [27]. The *AUCE_drs_* for the 22 patients during PS and NAVA are shown in Table 1. The *E_drs_* trajectories and trends for patients ventilated with PS are significantly different from those seen in patients ventilated using NAVA (p < 0.05 for 15/22 patients). All patients *E_drs_* trajectories are also included in S1 Fig.

**Table 1:** *AUCE_drs_* (5^th^, 25^th^, 50^th^, 75^th^, 95^th^ percentile) comparing PS and NAVA.

**Patient**	***AUCE_drs_* (*cmH2Os/l*)**											
	**PS**						**NAVA**					
	**5^th^**	**25^th^**	**50^th^**	**75^th^**	**95^th^**		**5^th^**	**25^th^**	**50^th^**	**75^th^**	**95^th^**	
**1**	27.2	28.6	29.5	30.6	32.4		11.2	14.0	16.4	18.7	23.5	+
**2**	6.2	8.9	10.5	12.2	17.3		-	3.5	4.1	8.4	17.0	
**3**	51.6	53.4	54.7	56.1	58.5		26.8	31.8	41.2	51.2	68.0	
**4**	23.0	28.0	31.5	35.8	41.1		14.1	15.8	18.2	21.4	26.2	+
**5**	38.1	40.7	42.9	45.2	48.5		16.1	19.6	22.1	24.9	29.0	+
**6**	55.5	60.9	64.5	69.0	78.4		28.3	44.3	53.6	62.9	79.8	
**7**	22.7	25.1	26.6	28.3	32.5		5.2	9.8	12.1	14.2	17.3	+
**8**	23.4	25.9	27.5	29.8	34.4		14.6	18.0	19.8	22.1	25.9	+
**9**	36.6	38.4	39.9	41.4	44.5		19.6	25.1	28.8	32.2	38.3	+
**10**	6.1	7.4	8.1	8.9	10.5		-	1.6	2.2	2.4	4.6	+
**11**	37.1	39.0	39.9	41.1	43.0		29.0	34.7	39.6	44.3	50.1	
**12**	22.4	25.3	27.8	32.9	48.4		5.7	8.1	10.5	15.6	28.1	
**13**	32.6	39.0	43.5	48.0	56.5		15.3	28.3	39.4	51.6	69.3	
**14**	30.9	37.2	41.5	47.9	60.8		14.5	18.9	23.0	28.6	46.4	
**15**	36.7	42.1	45.2	48.9	55.7		32.6	42.5	48.5	55.0	73.1	
**16**	24.2	26.3	27.6	29.1	31.3		8.6	10.4	11.9	13.7	18.2	+
**17**	20.4	24.8	28.0	30.8	35.2		8.0	12.5	15.3	18.6	24.4	+
**18**	43.9	48.3	50.0	51.8	55.8		27.3	37.0	43.5	51.3	66.8	
**19**	32.1	39.6	44.9	53.9	77.5		45.8	61.8	71.4	80.6	95.9	
**20**	34.3	37.9	40.1	42.4	46.8		23.6	29.4	33.0	36.7	42.8	
**21**	5.1	6.3	9.2	55.5	65.7		2.1	6.3	9.0	11.6	16.6	
**22**	23.8	34.4	38.5	40.9	44.0		31.3	41.6	47.9	56.5	68.7	
**Median**	29.1	35.8	39.2	41.3	45.7		15.7	19.3	22.6	26.8	33.7	+
**25^th^prct**	22.7	25.3	27.6	30.6	34.4		9.9	10.4	12.1	15.6	23.5	
**75^th^prct**	36.7	39.6	43.5	48.9	56.5		27.8	34.7	41.2	51.3	68.0	

prct: percentile; +: p < 0.05 when *AUCE_drs_* for PS is compared to NAVA *AUCE_drs_*

## Discussion

In this study, it was observed that all patients had negative *E_drs_* at the start of breathing as shown in Figs. 2 and 3. Negative *E_drs_* occurs when negative pressure is generated in patient’s pleural space causing air volume to enter the lung. During partially ventilation, the negativity is detected and the ventilator is trigger to provide positive pressure support. As lung volume increases due to positive pressure ventilation, *E_drs_* increases above 0, as expected clinically and from the model definition. As patient inspiratory demand is met, the magnitude of the *E_demand_* component of *E_drs_* reduces toward zero, as seen in *E_drs_* as a surrogate, and *E_drs_* becomes more positive.

Fundamentally, this extended model is thus general over SB and sedated MV patients, and implies that negative pressure ventilation will generate *E_drs_* < 0, while positive pressure ventilation will result in *E_drs_* > 0. Thus, the *E_drs_* can be used as a simple, real-time indicator to assess patients-specific disease state and response to MV. Equally, as *E_drs_* rises, it can be an indication of the changes in SB patients’ disease state and reduced demand.

For a fully sedated patient, the time-varying *E_drs_* values were found to be positive (*E_drs_* > 0) throughout the entire breath [22, 23, 28]. This outcome is consistent with what we would expect for a patient who is not providing the negative pleural pressure that facilitates spontaneous breathing. For SB patients who have their own inspiratory effort, *E_demand_* will be negative, lowering the overall *E_drs_* towards zero or to less than zero. More specifically, *E_drs_* will be less than zero when patient breathing demand is high at the beginning of inspiration, and will gradually decrease in magnitude as patient demand decreases during the breath.

An *E_drs_* > 0 implies that the positive pressure ventilation contributes or adds to the patient-specific lung elastance. Therefore, *E_drs_* > 0 is a measure of patient lung condition and response to MV. Only *E_drs_* > 0 may be considered as a potentially ‘harmful’ state to the lung, depending on level and trend throughout the breath. In particular, elastance is defined as pressure response to the delivered volume. High elastance (*E_drs_*) indicates more pressure per unit volume delivered, and thus greater risk for lesser volume and recruitment.

### *E_drs_* Trajectories and *AUCE_drs_*: Comparison between NAVA and PS

From Fig. 2, it is found that the shape of the *E_drs_* mapping for PS is different from NAVA. During PS, it is observed that the *E_drs_* mapping is more consistent and uniformly shaped in comparison to NAVA. This result indicates that different MV modes, or, more specifically, different pressure delivery techniques, will result in different *E_drs_* trajectories, as might be expected. In particular, the uniformity of *E_drs_* mapping observed during PS suggested lower breath variability compared to NAVA [26, 27]. Hence, these shapes and their *AUCE_drs_* (after 0.3 second normalised), can be monitored and modified to obtain lower, more desirable *E_drs_* to optimise MV delivery. In any of these cases, higher *E_drs_* may thus indicate greater lung damage and hence, greater risk for lung to overstretch [22].

In the cohort, it was found that the 5^th^-95^th^ percentile range for *E_drs_* was typically wider in NAVA than in PS, occurring in 20 out of 22 patients (p < 0.05). Figs. 2 and 3 clearly show more variation between breaths in NAVA mode compared to PS mode. This difference is as expected due to more variable pressure delivery in NAVA. The underlying method used by PS leads to the smooth, consistent curves seen in Figs. 2 and 3, while NAVA is dependent on the patient *Eadi*, which leads to much more variation in *E_drs_* between breaths, as seen in other studies comparing the matching of *Eadi* demand to tidal volume for these patients [26, 27].

Table 1 shows the *AUCE_drs_* for the 22 patients during PS and NAVA. The *AUCE_drs_* is the normalised area under the curve and can be used to describe patient-specific disease state similar to conventional two point static elastance [29]. The 95^th^ percentile *AUCE_drs_* was above 25 *cmH_2_Os/l* for 20 of 22 patients in PS mode, and only 15 of 22 patients in NAVA mode. Acute respiratory distress syndrome (ARDS) patients have been shown to have higher respiratory system elastance with *E_drs_* ≥ 25 *cmH_2_O/l* [30]. This result shows that, in most cases, the proposed *AUCE_drs_* is able to capture mechanics similar to those observed in an ARDS patient during full sedation and MV, giving confidence of the clinical relevance of the *AUCE_drs_* value. The results also show differences between modes and delivery of pressure on patient-specific response and risk. *AUCE_drs_* < 25 *cmH2Os/l* suggests that the patients’ lung in this SB study is more compliant than that of fully sedated ARDS patient lungs, as might be expected for SB patients.

### General Utility of Time-Varying *E_drs_*

Time-varying *E_drs_* is a measure of patient-specific response towards the ventilator [22]. Titrating care using this unique and physiologically relevant overall elastance parameter can potentially optimise both pneumatic settings of the ventilator (pressure and volume) simultaneously, as it incorporates both elements in its definition. It is a unique metric in capturing the relationship between pressure and (delivered) volume, compared to other approaches that try to titrate care in just one of these metrics (pressure or volume only).

Equally, the *AUCE_drs_* is able to capture a unique parameter that is directly relevant to respiratory mechanics of SB patients without the use of invasive oesophageal pressure measurements [4]. The application of *E_drs_* can potentially be used to guide PEEP selection, optimal pressure support and NAVA level in SB patients, which is currently not available without these additional invasive manoeuvres [4, 8]. This extended model and proof of concept analysis should thus open up new options in selecting the proper SB modes, and their associated PEEP or level of pressure support, as well as being general to both SB and fully sedated MV patients.

### Limitations

**Fig 4:**
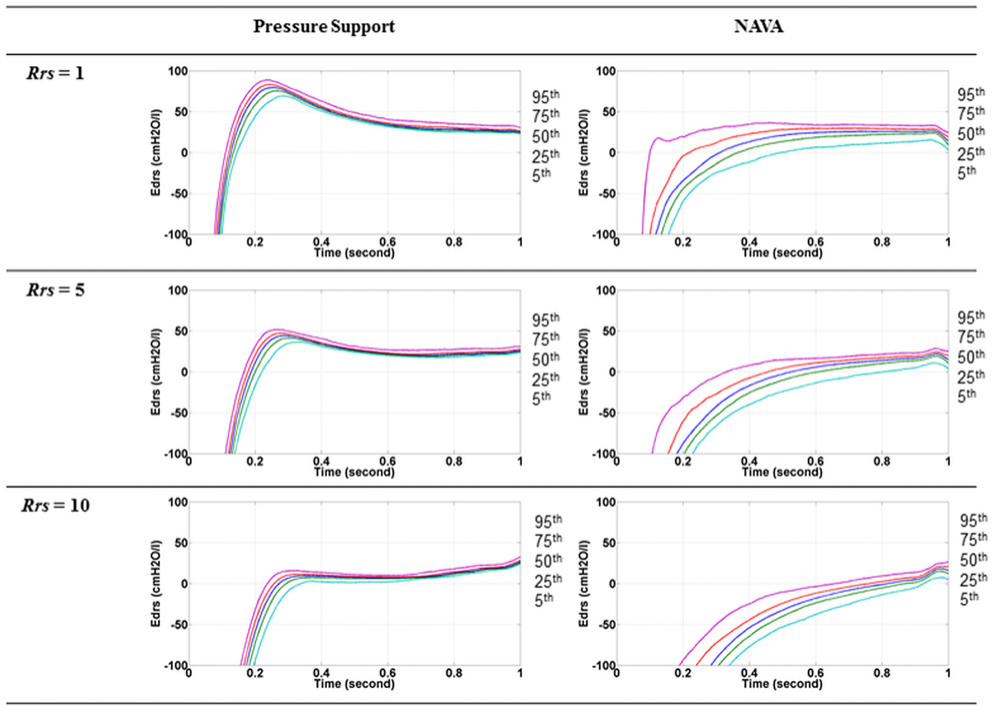
Time-varying *E_drs_* for Patient IV7 during PS (left) and NAVA (Right) at different airway resistance. The lines indicate the 5^th^ (Light blue), 25^th^ (Green) 50^th^ (Blue), 75^th^ (Red) and 95^th^ (Pink) percentile of all breathing cycles. The sequence where 5^th^, 25^th^, 50^th^, 75^th^ and 95^th^ percentile line occurs is labelled at the side of each figure.

**Airway Resistance Estimation**. One of the limitations of this study is the use of a constant resistance of 5 *cmH_2_Os/l*. As the estimation of *E_drs_* is dependent on the airway resistance (*R_rs_*), a constant resistance could yield incorrect *E_drs_* estimation. However, during intra-patient comparisons that switch between ventilation modes, the impact of *R_rs_* can be neglected in favour of trends. To quantify the impact, an example of the influence of different values of *R_rs_* (*R_rs_* = 1, 5, 10 *cmH_2_Os/l*) on the resulting *E_drs_* is shown in Fig. 4. A higher assumed resistance would shift the *E_drs_* trends downwards, whereas a lower assumed resistance would shift the *E_drs_* curves upwards. In addition, for some cases, the resistance value is unidentifiable due to unexpected features in the flow waveform [17]. By setting the resistance to a constant value, this issue can be avoided and the identifiability and identification quality improved. Thus, holding resistance constant at 5 *cmH_2_Os/l* across the cohort ensures equal comparison between and within patients. Equally, during intubation, the major component that attributes to the airway resistance is the endotracheal tube. Knowing the length and dimension of the tube may give an approximation to the airway resistance constant setting in favour of *E_drs_* trend comparison. From this estimation, the same procedures would follow and thus the methods presented are generalisable.

**Negative Elastance**. It is important to note that there is no physical ‘negative elastance’. The negative elastance can imply an unstable system or unstable due to external input of energy. The patient on fully controlled ventilation have showed only positive respiratory elastance [22, 23, 28]. Thus, the major negativity in elastance captured in this study, is due to patient input or breathing effort. The *E_demand_* proposed in this study is a construct, which aimed to capture the model instability, describing a condition where air volume enters the lung through a negative pressure generated by the patient during spontaneous breathing. Negative elastance has been used in the past to describe the in dynamic instability of the lung [31, 32]. This study aims to extend this concept to capture respiratory mechanics of a spontaneously patient by capturing the patient breathing effort.

**Time-Varying Elastance**. Time-varying *E_drs_* is not normally calculated in MV patients. It is a concept that provides unique information to monitor the patient-specific disease state and response to MV [33, 34]. When applied in SB patients, negative *E_drs_* values only correspond to the negative pressure generated in the pleural space to inflate the lung. Existing data on time-varying *E_drs_* or compliance in fully sedated MV patients has been shown to be positive [22, 23, 28]. *E_drs_* < 0 is only possible for patients who are breathing spontaneously, as it requires that the patient produces inspiratory effort. The validity of the estimated negative *E_drs_* as a measure of patient-specific demand similar to the use of oesophageal pressure in SB patients warrants further investigation, as the data and results presented in this study do not provide this opportunity. However, it should be noted that these measurements are not normally clinically available, limiting any clinical use.

All three elastance components (*E_lung_*, *E_chest_* and *E_demand_*) can be uniquely identified with several additional measurements and/or invasive procedures. During mechanical ventilation under full controlled mode (when the patient is paralysed), there is no influence in *E_demand_* and this term can be omitted. At this point, oesophageal pressure measurement can be used as a surrogate of pleural pressure [4] and thus, the patient-specific constant chest elastance, *E_chest_* can be estimated. At the same time, *E_lung_* can be estimated with the airway pressure and flow. With both known *E_lung_* and *E_chest_* values, the *E_demand_* during spontaneous breathing can be estimated. Thus, the approach presented here lumps these parameters into a single value identifiable from clinically available and common measurements, but notes how its shape is influenced by the individual components that comprise this value.

## Conclusions

A new model that defines conventional respiratory elastance into 3 different components is presented. The proposed model was able to capture unique dynamic respiratory mechanics for spontaneously breathing patients during PS and NAVA, which is otherwise not possible without added invasive manoeuvres that interrupt conventional care. The work presented here is the first of its kind to present a method and monitor time-varying *E_drs_* in SB patients without additional measuring equipment or interruption of care. It is a fully general model that is applicable to all MV modes and conditions.

## Supporting Information

S1 Fig Time-varying *E_drs_*, pressure, volume and electrical diaphragm activity (*Eadi*) curves for all patients included in the study during PS (left) and NAVA (Right). The lines indicate the 5^th^ (Light blue), 25^th^ (Green) 50^th^ (Blue), 75^th^ (Red) and 95^th^ (Pink) percentile of all breathing cycles. The sequence where 5^th^, 25^th^, 50^th^, 75^th^ and 95^th^ percentile line occurs is labelled at the side of each figure.
(DOCX)
