# Supplementary material for: Time-Varying Respiratory System Elastance: A Physiological Model for Patients Who Are Spontaneously Breathing
Source: PLoS One. 2015 Jan 22;10(1):e0114847. doi: 10.1371/journal.pone.0114847 (PMC4303416; doi:10.1371/journal.pone.0114847)
Supplement: S1 Fig — The lines indicate the 5th (Light blue), 25th (Green) 50th (Blue), 75th (Red) and 95th (Pink) percentile of all breathing cycles. The sequence where 5th, 25th, 50th, 75th and 95th percentile line occurs is labelled at the side of each figure. (DOCX) [file pone.0114847.s001.docx]

**Additional File Figure S1**

Figure S1 shows *E_drs_* trajectories for all patients included for the study. The axis are adjusted to the same range to allow inter-patient comparison. The data used for this study will be made available on request. Please contact [yeongshiong.chiew@canterbury.ac.nz](mailto:yeongshiong.chiew@canterbury.ac.nz) for further information.

| **Patient** | **Pressure Support (PS)** | **Neurally Adjusted Ventilatory Assist (NAVA)** |
| --- | --- | --- |
| **1** | 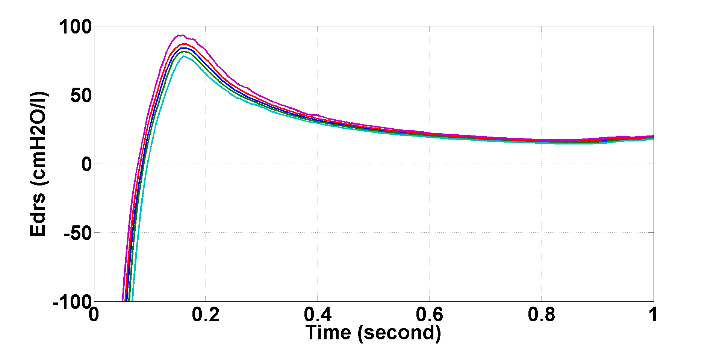 | 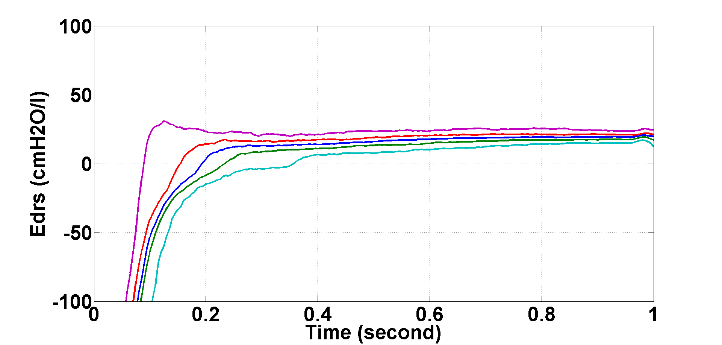 |
| **2** | 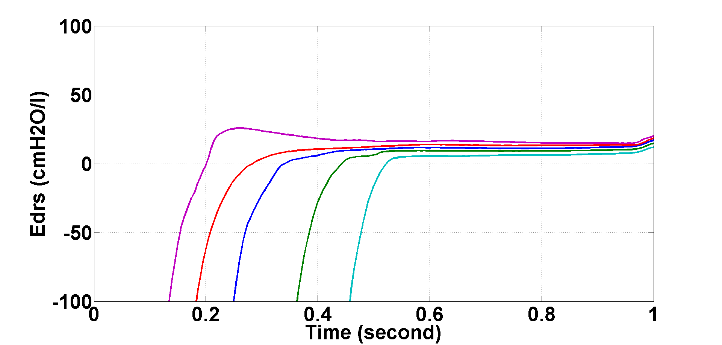 | 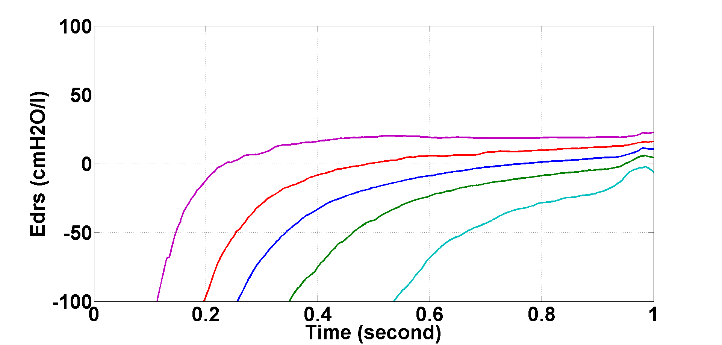 |
| **3** | 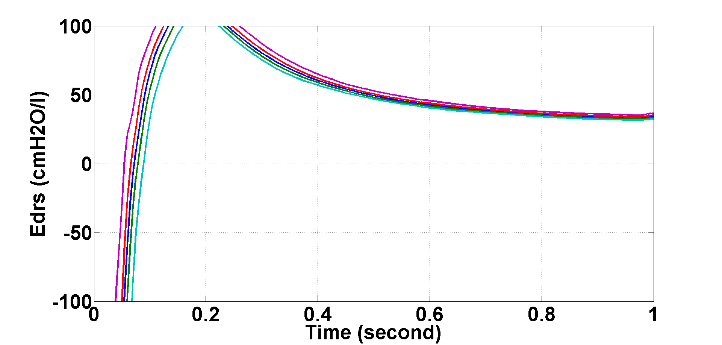 | 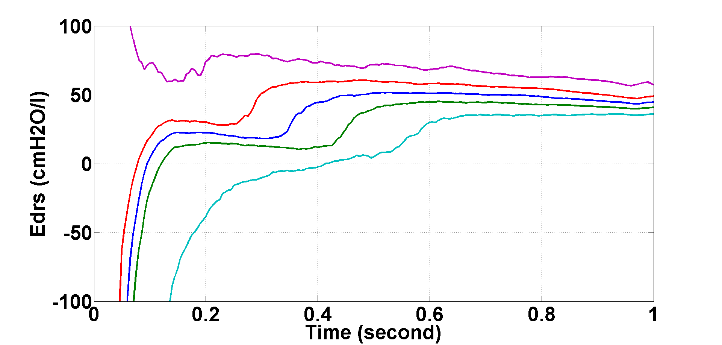 |
| **4** | 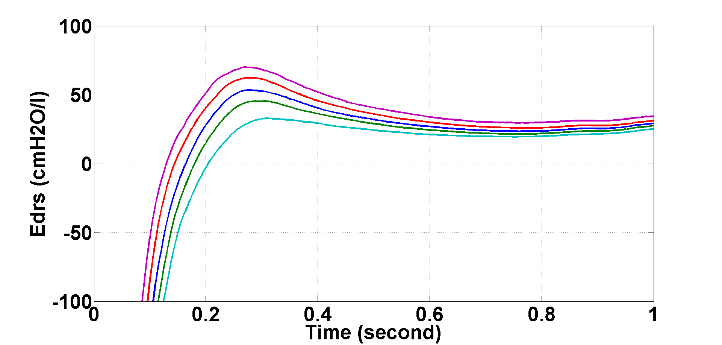 | 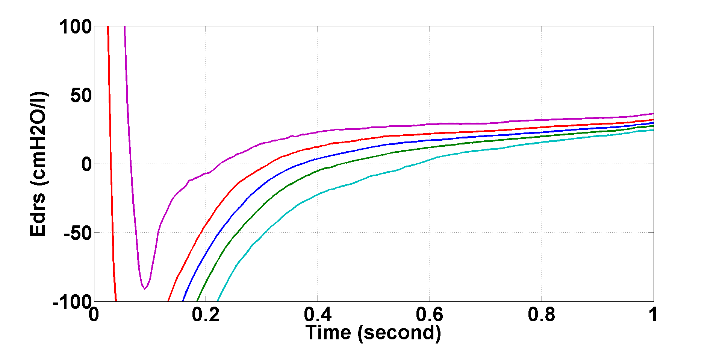 |
| **5** | 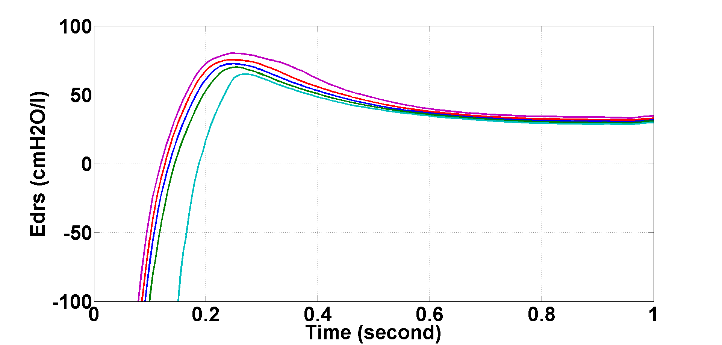 | 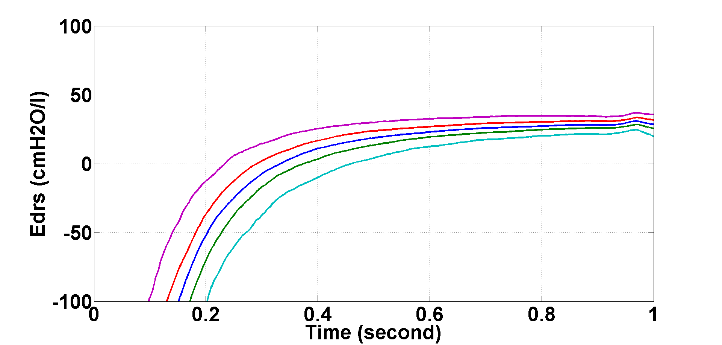 |
| **6** | 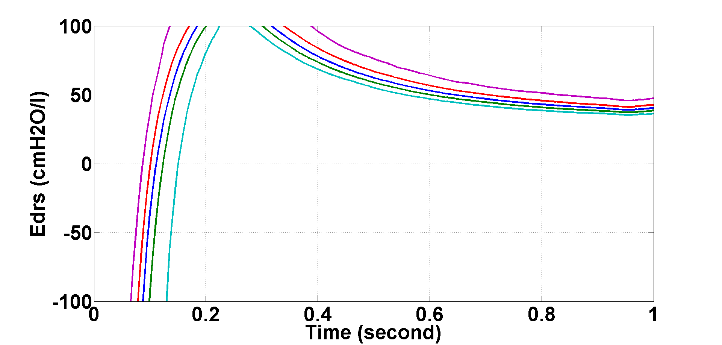 | 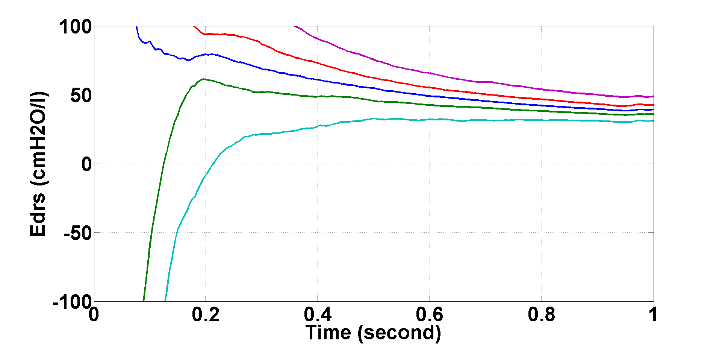 |
| **7** | 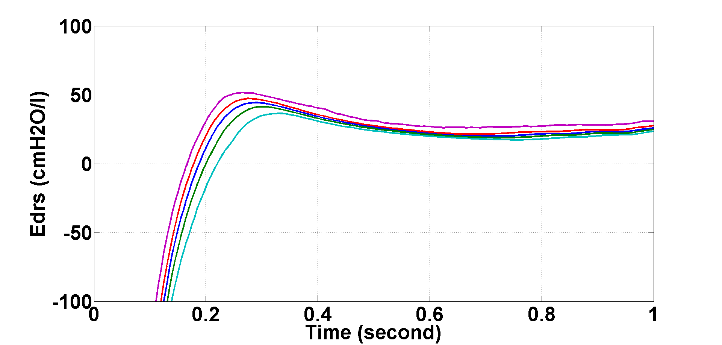 | 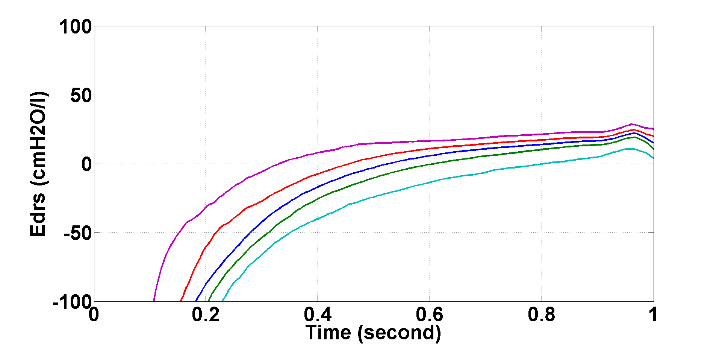 |
| **8** | 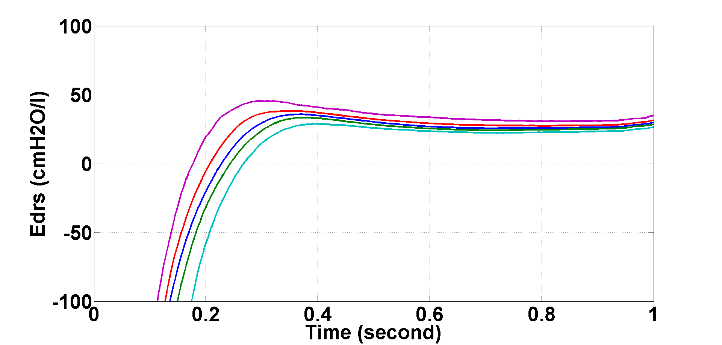 | 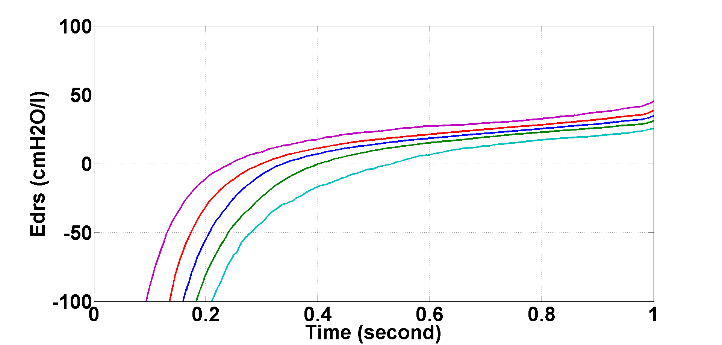 |
| **9** | 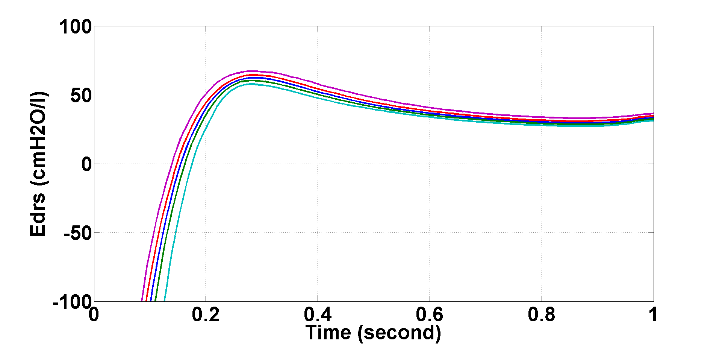 | 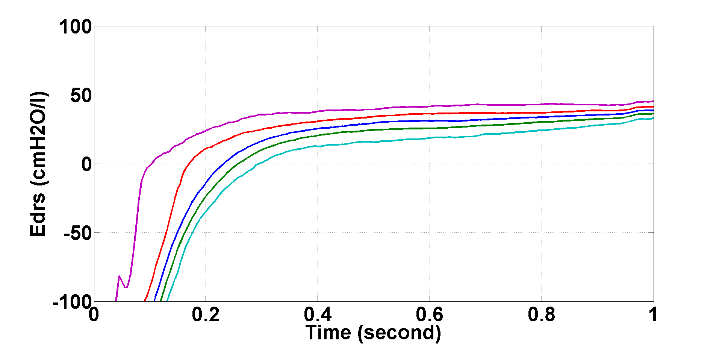 |
| **10** | 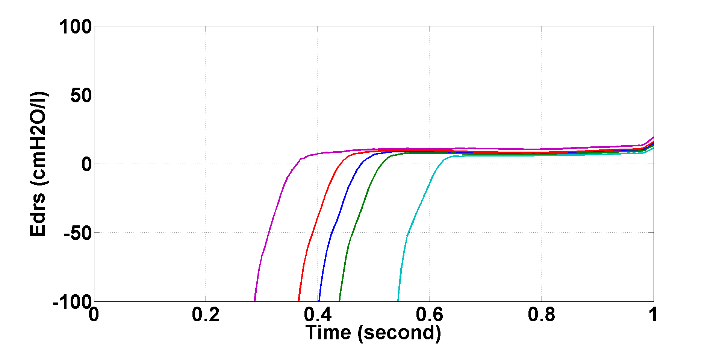 | 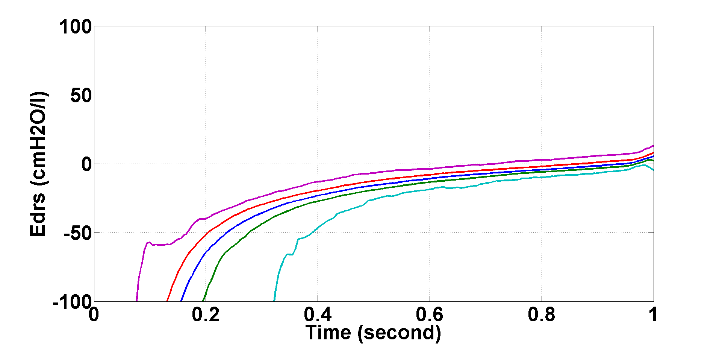 |
| **11** | 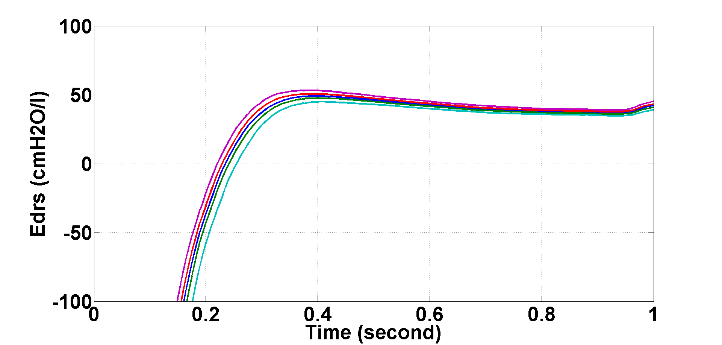 | 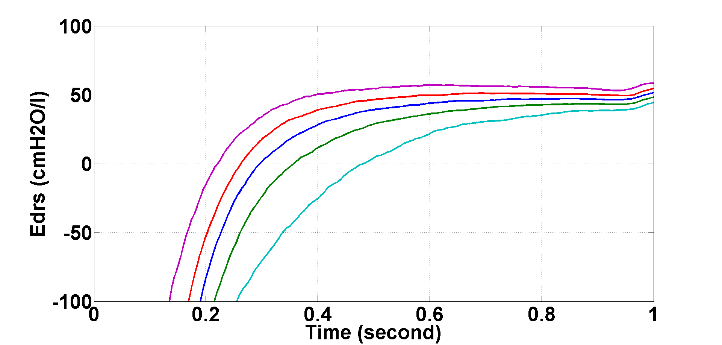 |
| **12** | 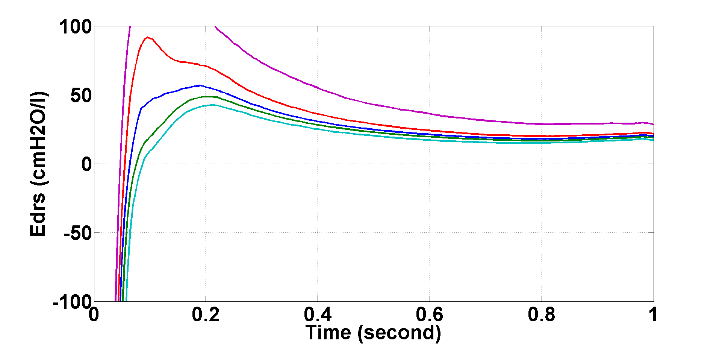 | 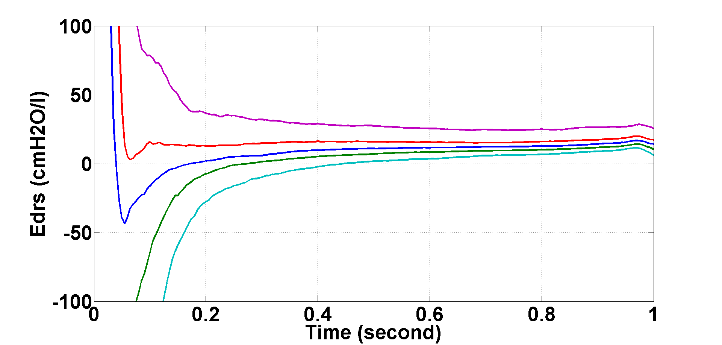 |
| **13** | 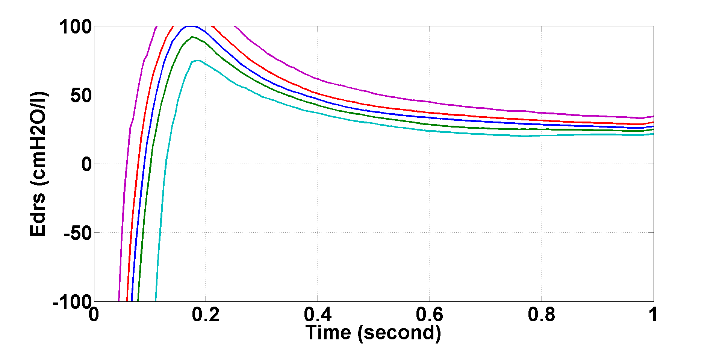 | 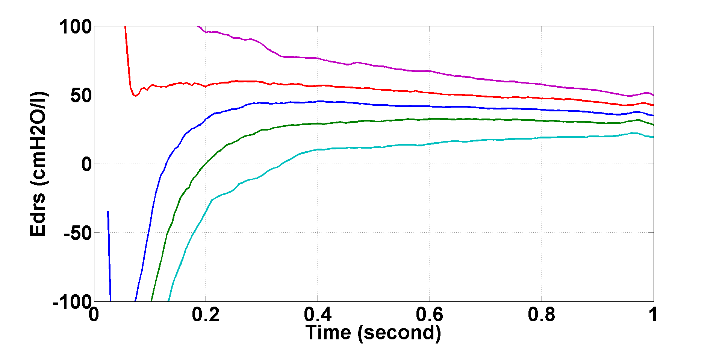 |
| **14** | 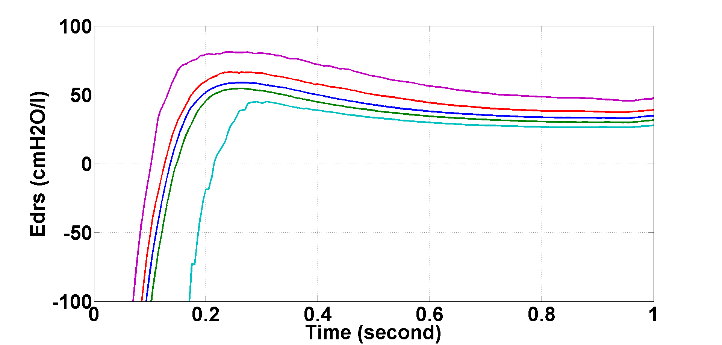 | 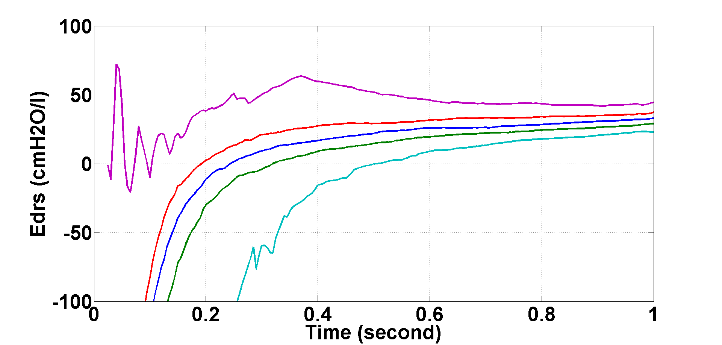 |
| **15** | 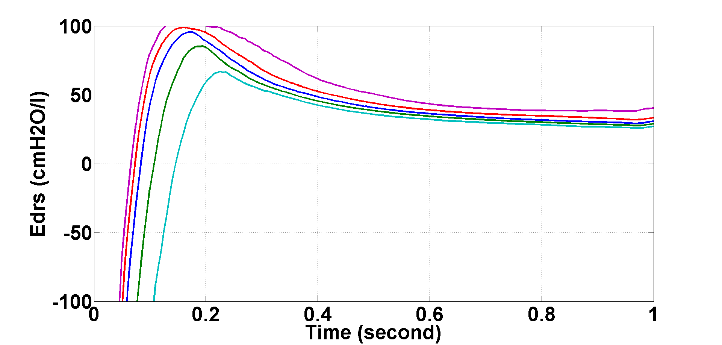 | 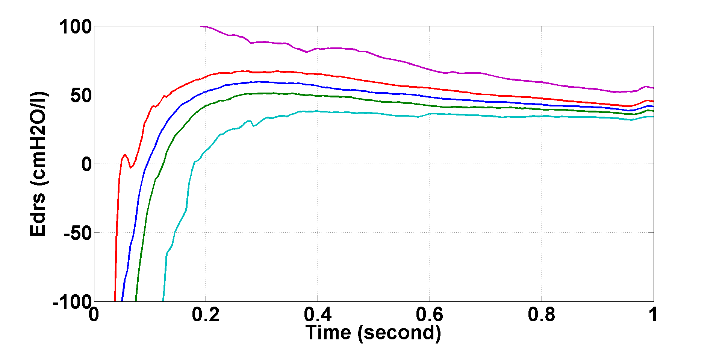 |
| **16** | 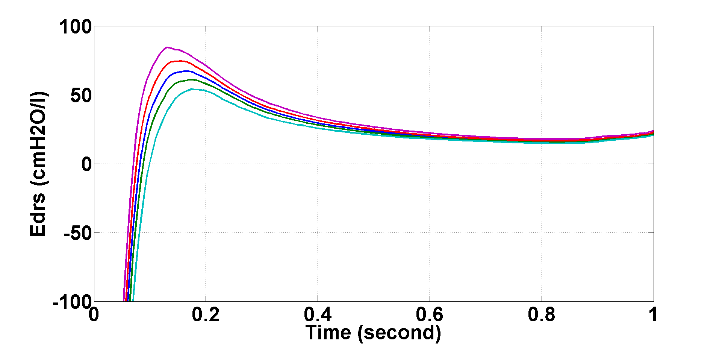 | 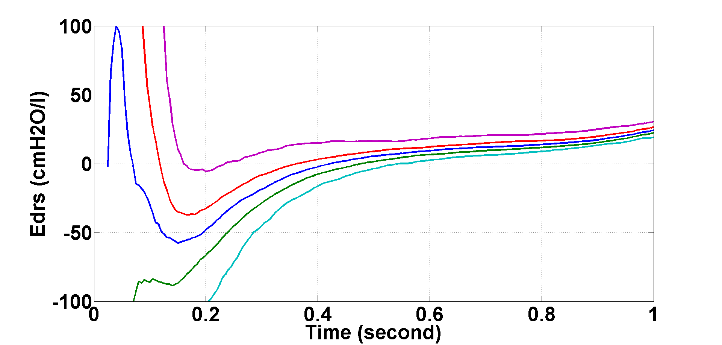 |
| **17** | 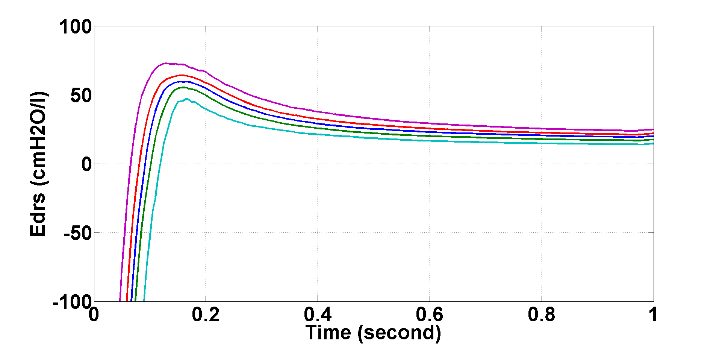 | 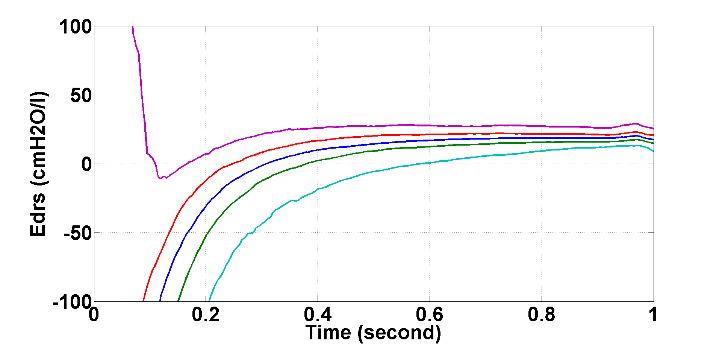 |
| **18** | 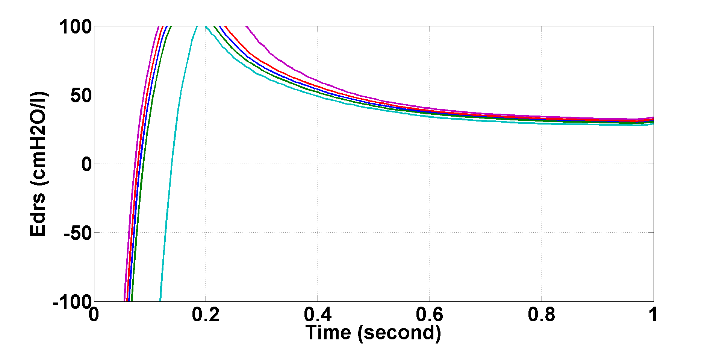 | 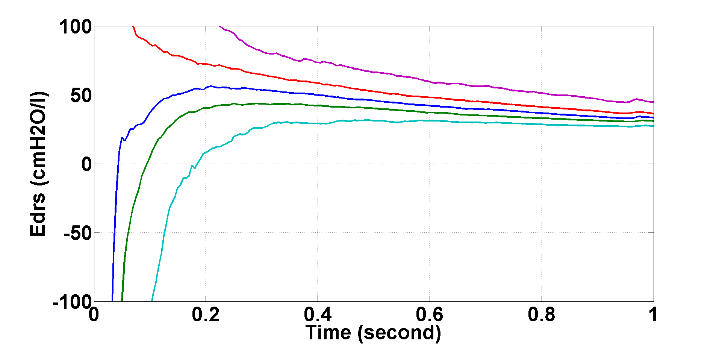 |
| **19** | 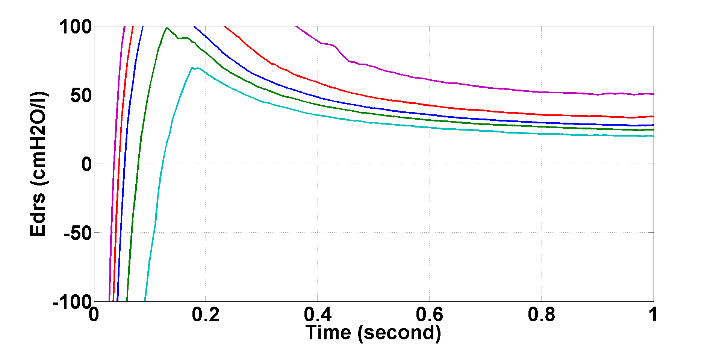 | 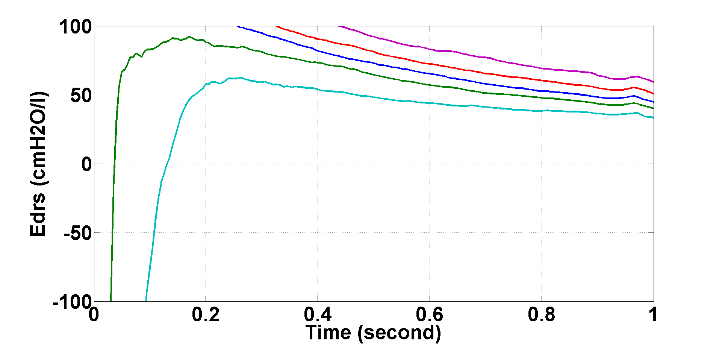 |
| **20** | 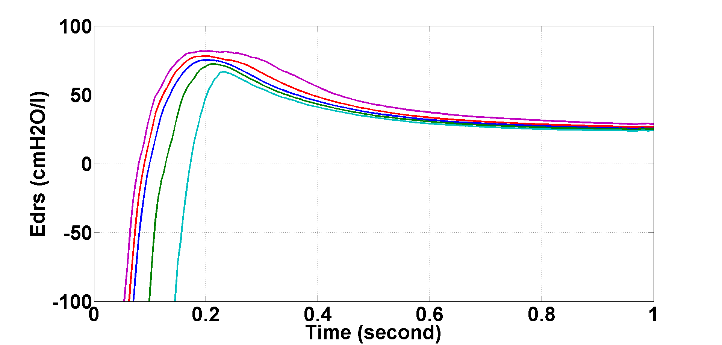 | 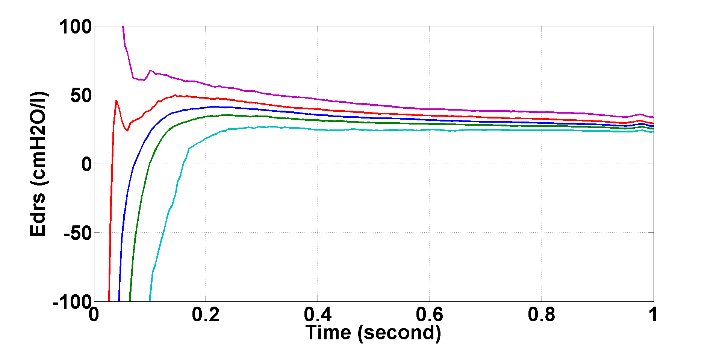 |
| **21** | 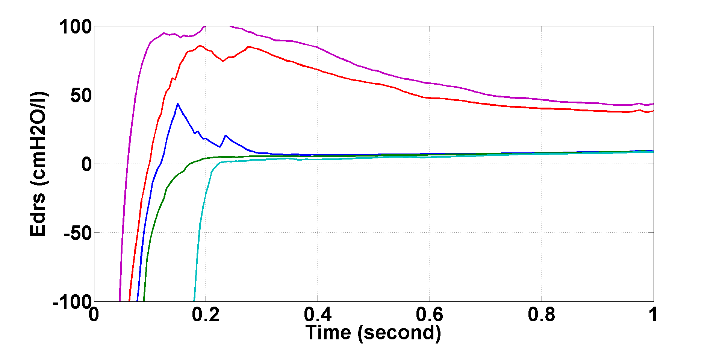 | 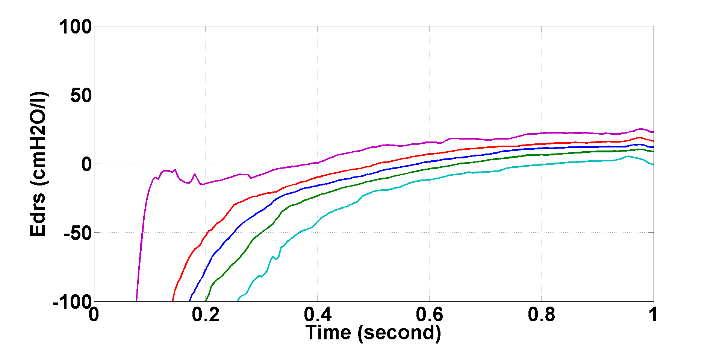 |
| **22** | 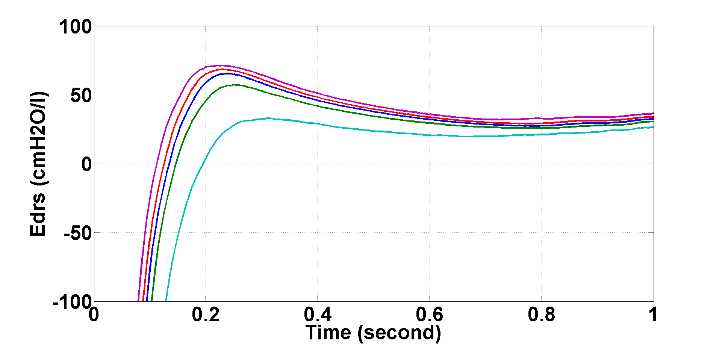 | 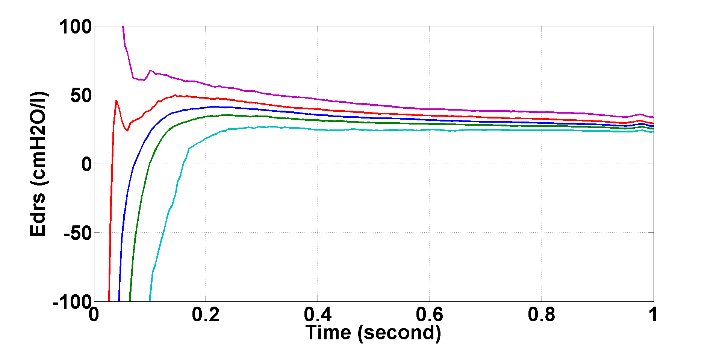 |

**Figure S1:** **Time-varying *E_drs_*, pressure, volume and electrical diaphragm activity (*Eadi*) curves for all patients during PS (left) and NAVA (Right).** The lines indicate the 5^th^ (Light blue), 25^th^ (Green) 50^th^ (Blue), 75^th^ (Red) and 95^th^ (Pink) percentile of all breathing cycles. The sequence where 5^th^, 25^th^, 50^th^, 75^th^ and 95^th^ percentile line occurs is labelled at the side of each figure.
